# Neurophysiology of Downhill Mountain Bike Athletes—Benchmark Assessments of Event-Related Potentials

**DOI:** 10.3390/s25175388

**Published:** 2025-09-01

**Authors:** Matthew G. Neill, Elizabeth K. S. Fletcher, Ember Larson, Kristina Fraser, Scott Ramsay, Jonathan D. Smirl, Carolyn A. Emery

**Affiliations:** 1 Sport Injury Prevention Research Centre, Faculty of Kinesiology, University of Calgary, Calgary, AB T2N 1N4, Canada; 2 Cerebrovascular Concussion Laboratory, Faculty of Kinesiology, University of Calgary, Calgary, AB T2N 1N4, Canada; 3 Alberta Children’s Hospital Research Institute, University of Calgary, Calgary, AB T2N 1N4, Canada; 4 Hotchkiss Brain Institute, University of Calgary, Calgary, AB T2N 1N4, Canada; 5 Human Performance Laboratory, Faculty of Kinesiology, University of Calgary, Calgary, AB T2N 1N4, Canada; 6 Libin Cardiovascular Institute of Alberta, University of Calgary, Calgary, AB T2N 1N4, Canada; 7 School of Nursing, University of British Columbia, Vancouver, BC V6T 1Z4, Canada; 8 British Columbia Children’s Hospital Research Institute, Provincial Health Services Authority, Vancouver, BC V6H 4C1, Canada

**Keywords:** event-related potentials, concussion, repetitive head impacts, mountain biking

## Abstract

**Background:** Downhill mountain biking (MTB) is a high-velocity sport where riders are potentially exposed to impacts and concussion. Rapidly obtained Event-Related Potentials (ERPs) are a promising use of portable electroencephalography for clinical assessment of concussion-related neurological impairment at the point of care. However, the baseline neuroelectric profile of this specific sporting population is not fully understood. This study investigated the amplitude and latency of the N100, P300 and N400 ERPs at baseline among MTB athletes by age, sex and caffeine consumption. **Methods:** MTB athletes, aged 13–45, competing in the 2024 Whistler Crankworx MTB competition were recruited for this cross-sectional analysis. Self-reported sex, age and caffeine consumption were captured and pre-event baseline neurological evaluations of the N100, P300 and N400 ERPs (*n* = 92) were completed using a portable electroencephalograph (NeuroCatch). **Results:** Females had a 1.12 µV higher N100 amplitude (95%CI; 0.15, 2.08, *p* = 0.024) and 2.11 µV higher P300 amplitude (95%CI; 0.69, 3.52, *p* = 0.004) than males. Further, the N400 amplitude was 0.05 µV lower per year that age increases (95%CI; −0.08, −0.01, *p* = 0.011). Greater than normal caffeine consumption was associated with a 1.39 µV lower (95%CI; −2.68, −0.09, *p* = 0.036) and 18.96 ms (−28.29, −9.64, *p* < 0.001) faster N100 response. **Conclusions:** Age, sex and caffeine consumption must be considered to inform the clinical implement of ERPs for monitoring concussion in extreme sport athletes.

## 1. Introduction

Downhill mountain biking is a popular sport that attracts participants who enjoy the challenge, adventure, physical activity and community that the sport provides [1]. However, downhill mountain biking is considerably injurious across the elite [2] and recreational [3] levels with sport-related concussion (SRC) being a common injury. Specifically, SRC is a prevalent injury in downhill mountain biking, representing 5–15% of all injuries [4]. Moreover, SRC is a functional injury that is not detected using standard medical neuroimaging assessments [5]. Thus, diagnosis and management of SRC relies on physician expertise in interpreting the mechanism of injury, clinical presentation and subjective symptom reporting of patients following SRC [5]. Given that downhill mountain biking crashes often result in head impacts (or body impacts with forces transmitted to the head), many crashes have a plausible mechanism for SRC. Furthermore, there is currently no downhill mountain biking-specific assessment for SRC [6]. This is concerning since as many as 68% of these riders continue riding after the onset of SRC symptoms following a crash [7]. Considering the potentially devastating consequences of a secondary head injury following SRC [8], there is a present need for point-of-care assessments that can support diagnosis and removal from play/riding for downhill mountain biking athletes who sustain an SRC following a crash. Moreover, even when downhill mountain bikers do not crash, they accumulate a high volume of repetitive head impacts (RHIs). There is evidence that the RHI exposure in a single downhill mountain bike run is comparable to a game of rugby [9,10]. Given the growing concern that exposure to these impacts are associated with neurological impairment [11,12], there is a present need to improve the quantification of the neurological changes associated with RHI. Furthermore, biological markers of acute concussive injury must be able to discriminate between the neurological consequences of acute concussion and chronic RHI exposure.

There is ongoing investigation of physiologically informed biological indicators of SRC including alterations in electroencephalography (EEG) [13], cerebral blood flow [14], blood biomarkers [15] and Event-Related Potentials (ERPs) [16]. ERPs are the quantification of neuro-electric responses to time-locked, standardised stimulus, measured using EEG. ERPs follow similar patterns, creating waveforms with recognisable peaks that can be quantified by their amplitude and latency. EEG is a longstanding technology, first developed in the 1924 [17,18], yet the clinical applications of ERPs are expanding. While time-consuming, signal processing, data filtering and peak-selection limited the clinical application of ERPs, advances in machine learning algorithms have allowed for ERPs to be obtained relatively rapidly using portable equipment [19,20]. Thus, ERP evaluations have become a feasible technology for point-of-care clinical application in a sporting environment [20].

There is evidence of various ERPs being sensitive to acute [21] and long-term changes in neurological function [16] following SRC. Moreover, studies have demonstrated that ERPs are sensitive to neurological changes associated with RHIs approximated by exposure to seasons of collision sport exposure [21,22] or helmet accelerometers [23]. Within-subject differences from baseline in processing performance of the N100, P300 and N400 responses have been observed following concussion and RHIs [16,21,22,23]. Thus, the present study investigates the N100, P300 and N400 ERPs. The N100 ERP occurs roughly 100 ms following a tone and is a measure of sensory neurological processing [24]. The P300 response occurs 300 ms following an oddball stimulus and approximates attentional performance [25,26]. The N400 ERP occurs roughly 400 ms following a semantic oddball stimulus and reflects cognitive-semantic neurological performance [27,28].

While optimising ERPs for clinical application, considerations in reducing testing procedure duration have limited the number of electrodes that can be feasibly included in the assessment. Thus, when EEG technology is optimised for clinical applications, spatial resolution is compromised. While High-Density EEG can employ up to 256 electrodes to quantify both the special and temporal ERP response, clinical EEG can use as few as 5 electrodes, including ground, reference and electrooculogram electrodes [20], quantifying a generalised neurological response. Thus, while some known confounding influences have been identified using HD EEG [29], the influence of these factors on clinical ERPs assessed only at the midline must also be explored.

ERPs differ based on a variety of individual factors, including age [30] and sex [22,31]. Moreover, recent evidence suggests that baseline ERPs differ between collision sport athletes and the general population [32], yet potential confounding factors have not been explored in athletic populations. Therefore, quantification of the ERP responses, and the relevant confounding influences of ERPs within a given sport is clinically essential to understand ERP outcomes measured following suspected SRC and sport-related RHI exposure.

Another important consideration for assessing ERPs in athletic populations is that participants may be trying to maximise neurological performance during competition, using performance enhancing substances, such as caffeine [33]. Caffeine is a compound known for physical and cognitive enhancement frequently used by athletes to improve sport performance [34,35,36,37]. There is evidence that caffeine alters the amplitude and latency of ERPs as well [35]. However, the clinical implications for caffeine consumption, over-consumption and withdrawal on ERPs in a sporting population have yet to be elucidated.

Accordingly, population-specific investigations of baseline ERP measures, including investigations of potential influences such as sex, age and caffeine consumption are necessary to support the clinical application of novel ERP assessments of neurological function in a particular sporting context. The purpose of this study was to characterise the mean resting neurological responses of downhill mountain-bikers using rapidly obtained N100, P300 and N400 ERPs. Moreover, a secondary aim of this study was to investigate the potential influence of sex, age and caffeine consumption on the N100, P300 and N400 ERPs in an exploratory analysis of downhill mountain bikers. It was hypothesised that downhill mountain bikers would elicit baseline ERPs similar to responses observed in collision sport athletes [22,32]. Moreover, it was expected that younger age and female sex would be associated with improved neurological performance (increased ERP amplitudes and reduced latencies). Furthermore, it is hypothesised that caffeine will generally be associated with improved neurological performance, and that withdrawal from caffeine will demonstrate the opposite association.

## 2. Materials and Methods

This cross-sectional study recruited downhill mountain bikers competing in an elite competition event (Crankworx, Whistler, BC, Canada, 2024) in alignment with the ‘Surveillance in Highschool and community sport to REDuce concussions and their consequences’ (SHRED Concussions) cohort study [38,39]. Participants were eligible to participate if they were competing in any event at the Crankworx competition and were at least 14 years of age. Participants were elite professional or semi-professional downhill mountain bikers competing in downhill race disciplines. While there were more males registered to compete in the various Whistler Crankworx downhill mountain biking events, participants of both sexes were recruited. Participants were excluded if they were not proficient in English, but those whose first language was not English were eligible to participate, provided they self-report proficiency in English. The study underwent ethics review by the University of Calgary Conjoint Health Research Ethics Board (CHREB ID: REB21-0968, REB20-1662).

This cross-sectional analysis assessed athletes at baseline, while they were checking in to the competition and receiving their race packages. Data was collected in a vacant conference room, or on site with a mobile laboratory (the SHRed mobile: https://www.ucalgary.ca/sport-injury-prevention-research-centre/shred-mobile, accessed on 25 August 2025) residing in a class A recreational vehicle that was positioned on the competition grounds.

### 2.1. Assessment of Exposures

Right before the completion of the neurological evaluation, caffeine consumption was assessed via ordinal self-report questionnaire whereby participants reported the number of caffeinated beverages they had consumed in the past 24 h as 0, 1–2, 3–4, or 5+. Precise dose of caffeine consumption was not collected; thus, caffeine consumption was dichotomised into ‘Yes’ and ‘No’ for those that did and did not consume caffeine. Furthermore, Participants ranked whether their caffeine consumption on the day of testing was more, less, or equal to their normal consumption. Self-report mood (very low, low, good, very good), sleep (<4 h, 4–6 h, 6–8 h, >8.h), alcohol consumption (yes/no), nicotine consumption (yes/no) and psychoactive consumption (yes/no) was also recorded. The self-report questionnaire was collected as part of the integrated assessment on the device that captured the ERPs, allowing for exposure variables to be collected concurrently with the ERPs.

### 2.2. Neurological Assessment

The benchmark neurological evaluation consisted of a 375 s auditory EEG assessment completed in a quiet indoor space. ERP outcomes were collected using a portable, point-of-care EEG system (NeuroCatch, Surrey, BV, Canada) employing 6 electrodes, with midline locations; Fz, Pz, Cz, as well as a ground electrode at Afz, an electrooculogram recorded from FPz, and a reference electrode on the right earlobe. This device was selected for this study as it is designed for application in point of care athletic settings [19,20,21,22,23]. Data was collected at a sampling rate of 500 Hz. Prior to assessment, the skin on the forehead and right ear was cleaned using isopropyl alcohol wipes (LernaPharm, Montreal, QC, Canada) and a mild abrasive gel (Nuprep, Weaver and Company, Aurora, CO, USA). Conductive gel (Signa Gel, Parker Labs, Fairfield, NJ, USA) was used to lower impedance between the electrodes and the scalp. An impedance < 25 KΩ was required for each electrode before commencing the evaluation. During the evaluation, the participant was instructed to focus on an eye-level fixation point roughly 2 m in front of them. Integrated headphones delivered the ERP auditory stimulus.

### 2.3. ERP Stimulus

A standardised auditory stimulus was used to quantify the N100, P300 and N400 responses. The stimulus consisted of a sequence of tones and narrated word pairs, in alternating order [20]. The series of tones comprised mostly standard tones (264; 91% of total) and infrequent oddball tones (24; 9% of total) to obtain the N100 (auditory sensation) and P300 (basic attention) ERP responses. The standard tones were 80 dB, while the oddball tones were 105 dB. The odd-ball paradigm [26] is the most validated way to elicit the p300 ERP. This paradigm is primarily attentional, whereas other paradigms, such as the go-no-go tasks require response inhibition and motor control [40]. Thus, when using a point of care tool to automate some of the processing, the odd ball paradigm elicits the easiest to select peak [20]. The odd ball paradigm has been used previously to assess attentional processing in athletes and is used clinically to assess for differences following brain injury. Lastly, the odd ball paradigm tasks are performed quickly, are not fatiguing and integrate well into the word-pair matching tasks used in the present assessment. The word pairs (72) were either semantically related, such as ‘pen’ and ‘paper’ (50%) or were semantically unrelated (50%) and used to obtain the N400 (cognitive processing) ERP. The EEG recording was time-locked to the stimulus delivered through the integrated headphones.

### 2.4. Data Processing

The NeuroCatch platform automatically applies a fourth order Butterworth filter, a notch filter and an adaptive filter that corrects for ocular artefacts. The NeuroCatch software (version 2.0) automatically normalises ERPs using detrending which accounts for drift between epochs. The data from each electrode and each trial was grand averaged to display a composite trace −100 ms to +900 ms from the stimulus. N100, P300 and N400 peaks are identified using a blinded rater. The N100 peak was selected as the largest negative deviation in the neuroelectric waveform between 50 and 150 ms [22]. The P300 was selected as the largest positive deviation in the neuroelectric waveform between 250 and 450 ms, occurring later than the standard P200 response [22]. In the case of a bifid peak, whereby separate P300 a and P300 b peaks of similar amplitude were available, the P300 a response was selected [41]. The N400 was selected as the largest negative deviation occurring roughly between 300 ms and 550 ms, occurring after the PNM response and bookended by the P200 and P600 responses [22]. The same peak was selected to estimate amplitude and latency for each ERP. Amplitude was estimated in reference to 0 μV, whereas latency was described in reference to stimulus onset (0 ms).

### 2.5. Statistical Anlaysis

All analysis was completed using Stata 18 (StataCorp. 2025. Stata Statistical Software: Release 18. College Station, TX, USA: StataCorp LLC). The threshold for statistical significance was set a priori at an alpha value of 0.05. Mean and 95% confidence intervals (95% CI) for participant demographics were calculated, stratified by sex. Mean and 95% CI were calculated for each ERP outcome. Bivariate linear regression models were fit between known confounding factors and each ERP amplitude and latency to assess if these known confounds were related to ERP outcomes in the present study. If these were statistically significant, they were included in the subsequent models. Six multivariable linear regression models were fit between age, sex, an age by sex interaction term and the six ERP outcomes (N100, P300, N400 amplitude and latency). Interaction terms were removed from the models if the Wald test for the interaction term was not statistically significant. Exploratory bivariate linear regression models were fit to explore the influence of caffeine consumption and deviations from normal caffeine consumption on the outcomes of N100, P300, N400 amplitude and latency. Model residuals were assessed to ensure normality and homoscedasticity.

## 3. Results

Ninety-two participants (66 male, 26 female) participated in the study. While 33 (24 male, 9 female) participants did not consume caffeine prior to testing, 59 (42 male, 17 female) did (Table 1). Mean and 95% Confidence Intervals (CIs) of the baseline ERP responses were calculated and stratified by sex (Table 2). Multivariable models fit with the interaction of age and sex did not reveal evidence of effect measure modification between age and sex on ERP outcomes (all *p* > 0.165). Thus, models without the interaction between age and sex were reported (Table 3). Females demonstrated a 1.15 μV higher amplitude N100 (95% CI: 0.20, 2.10, *p* = 0.019) and 1.89 μV higher amplitude P300 (95% CI: 0.48, 3.29, *p* = 0.009) response compared to males (Table 3). Age was associated with a −0.06 μV decrease in the amplitude of the N400 response per year of age (95% CI: −0.10, −0.02, *p* = 0.003, Table 3). Caffeine consumption was not associated with difference in any of the ERP amplitude or latencies (all *p* ≥ 0.328, Table 4). The N100 was 1.39 μV smaller (95% CI: −2.68, −0.09, *p* = 0.036) in amplitude and occurred 18.96 ms earlier (95% CI: −28.29, −9.64, *p* < 0.001, Table 4**)** in participants that consumed more caffeine that usual before assessment. Bivariate linear regression models investigating the potential influence of alcohol, nicotine and psychoactive consumption and mood on the results were included as Supplementary Material.

## 4. Discussion

The purpose of this study was to establish the baseline neuroelectric profile of competition downhill mountain bike riders, and investigate the potential influences of age, sex and caffeine on the N100, P300 and N400 ERPs. The key findings from this study were that female downhill mountain bikers had higher N100 and P300 amplitudes compared to males and that the amplitudes of the N400 ERP decreased with increasing age in downhill mountain bikers. There was evidence that those who consumed more caffeine compared to their normal consumption had faster, but smaller amplitude N100 responses compared to those who consumed their regular amount based on self-report.

In the present study, the estimated mean N100 amplitudes were 4.49 μV for male and 5.64 μV for female downhill mountain biking athletes (Table 2). Previous research using the same ERP assessment technique observed that participants of ‘high-contact’ sport display larger amplitude N100 responses compared to ‘low-contact sport’, observing N100 amplitudes of 4.60 μV and 3.15 μV, respectively [22]. Previous studies have also detected differences in N400 responses between high-contact athletes and controls, with one detecting longer latency in the high-contact group [22], and another detecting smaller amplitudes in mixed martial artists relative to controls [32]. The present study estimated the mean N400 latency was 414.15 ms for male and 430.08 ms for female mountain bikers (Table 2). These estimates are similar to the mean 433.50 ms N400 latency observed in high-contact athletes, which was higher than the 359.16 ms observed in the low-contact group [22]. Taken together, similarities in the N100 and N400 responses observed in the present study and previous research may indicate that the chronic neurological impact of downhill mountain bike riding are consistent with that of other high-contact/-collision sports, such as rugby, ice hockey and soccer.

There is evidence that downhill mountain biking athletes sustain between 18 and 45 10 g or higher RHIs per ride, depending on the course being ridden [9]. This is similar to the levels of head acceleration event exposure in one h of rugby match play [10]. Thus, the similar baseline neurological processing levels observed between the present study and prior research in the N100 and N400 ERPs may be related to the RHIs associated with collision sport participation. However, while a season of contact sport exposure has been demonstrated to increase N400 latency relative to preseason baseline in youth hockey and football players [21,23], it remains unclear if N400 performance recovers during off-season, or remains chronically depressed. Future research should investigate if downhill mountain biking exposure elicits similar changes in ERP outcomes.

While there is evidence that the relationship between collision sport exposure and ERPs is not modified by sex [22], questions remain on the influence of sex on ERP responses irrespective of sport participation. Some studies demonstrate that females elicit more latent, lower amplitude P300 responses than males [31], while others find that females elicit larger amplitude P300 responses [42]. There is also evidence that females elicit lower amplitude N400 responses, compared to males [22,29,43]. While discrepancies may be the result of methodological and technical differences, it is imperative that the influence of sex on ERPs derived using clinical point of care assessments is understood to inform clinical interpretations. A previous study observed that the amplitude of the P300 response is higher in females compared to males using the same assessment technique as the present study with a comparable sample size (*n* = 89) [22]. Conversely, our study detected a higher N100 amplitude in female participants relative to males, while the aforementioned study did not detect a significantly different N100 between sexes [22]. Moreover, the previous study identified that females exhibited a larger amplitude N400 response that was not observed in the present study. In comparison to the current participants (Table 1), the study by D’Arcy and colleagues used a more homogenous sample of 89 high school student athletes (age 15–17 years) attending the same private boarding school. Thus, differences in sample age, background, education level and sport may explain some of the discrepant sex-difference findings in the present study [29].

Furthermore, there is evidence that the P300 ERP is altered across the lifespan in accordance with neurodevelopmental trajectories, though estimates of the association vary depending on the techniques used to elicit the P300 [30]. While the present study did not detect age-related changes in the N100 and P300 responses, we did observe a significant reduction in N400 amplitude associated with older age. The N400 is representative of cognitive processing performance, perhaps indicating that the N400 is sensitive to age-associated cognitive decline, although other studies have not detected this potential association [29]. The sample of the present study did not include participants older than 45 years of age, perhaps limiting the detection of age-related neurological impairment that occurs in older age [44]. Moreover, engagement in physical activity (such as participation in sport) is known to attenuate the physiological ageing of the brain [45,46]. Thus, even older participants in the present study may exhibit the neurological performance of younger, less active member of the general population [46]. This is supported by evidence that P300 amplitudes are higher in fitter compared to ‘less-fit’ age-matched comparators [47].

While the present study found evidence that age and sex are relevant to the clinical interpretation of ERPs, the clinical application of neuroimaging technology in sport must also consider the potential of lifestyle factors to confound assessments. There is mixed evidence regarding the effect of caffeine consumption on neurological processing performance. Previous research has demonstrated that caffeine consumption generally improves the P300 and N100 responses [35,48], with larger effects being observed when the timing of the ERP stimulus is unpredictable [35,49]. Moreover, 200 mg of caffeine consumption is associated with an attenuation of the P300 amplitude decline following fatiguing exercise in taekwondo athletes [50]. Conversely, caffeine withdrawal in habitual users is associated with an acute decline in P300 amplitude each day of caffeine abstinence, but without changes in latency [51]. In the present study, there were no differences in ERP responses between those who did and did not consume caffeine. However, the N100 response was significantly reduced in amplitude and latency in those who consumed more caffeine than usual compared to those who consumed an amount of caffeine closer to their normal daily consumption. Thus, the results from the present study indicate that deviation from habitual caffeine use may acutely influence the P300 in this population. The precise mechanisms by which an increase from habitual use in caffeine consumption influences neurological processing performance is unclear. There is evidence that caffeine alters neurovascular coupling [52]. and reduces resting cerebral blood flow [53,54], two mechanisms related to the pathophysiology of concussion [14]. Moreover, direct effects of caffeine on the nervous system appear to increase the frequency bands of neural activation which may influence ERPs and exacerbate the neurometabolic cascade of concussion [55,56]. The data in the present study was based upon self-reported values and did not alter caffeine consumption in participants. Thus, for many, the observed abstinence from- or consumption of caffeine may have been typical for the preparation phase for participation in a competitive event, but not typical relative to daily use outside of competition. Thus, a new homeostatic baseline may have been achieved, consistent with evidence of tolerance changes to repeated caffeine consumption [57]. Given that self-reported regular caffeine use was not associated with differences in ERP outcomes, the evidence from this study suggests that encouraging participants to engage in typical caffeine consumption may be preferred to restricting caffeine use prior to neurophysiological testing.

The present study is not without limitations. This study investigated demographic and lifestyle factors of interest when performing point of care clinical ERP assessment in athletes. The cross-sectional design of the study is not able to inform causal inferences between age, sex or caffeine consumption and ERPs. Future research may better address the influence of age on ERPs by tracking a cohort of participants over time and better investigate the influence of caffeine on ERPs by experimentally modifying caffeine consumption by participants. Furthermore, the sample of the present study included elite downhill mountain biking athletes. Thus, it is expected that the athletes had extensive history of downhill riding and a history of concussion and musculoskeletal injury consistent with other research in downhill mountain biking [4,7], yet injury and sport participation history were not explicitly assessed.

The assessment technique in the present study limited the number and location of electrodes, but showed versatility to be used in a point-of-care setting at an outdoor sporting event. While pre-frontal electrodes are suitable for assessing global N100, P300 and N400 responses, high-density EEG techniques provide better special resolution, improving detection of subtle regional differences in neural activation. However, this was also a strength of the study, as it allows the findings to be generalizable to clinical ERP applications. Moreover, the present study used an ERP technique which determines the amplitude and latency of a grand average response calculated from numerous repeated trials. While this is consistent with clinical applications of ERP evaluations, grand averaging of ERP waveforms prior to selecting peaks limits interpretation, especially in regard to changes in amplitude. When an ERP latency has high variability between trials, amplitude of the grand average wave form will be smaller than the mean amplitude of the peaks of each trial. Thus, the interpretations of lower amplitude ERP responses cannot distinguish between a temporally stable yet small magnitude response and a highly temporally variable yet unchanged magnitude response [58]. Thus, in a clinical application, while changes in grand average ERP amplitude may detect neurological dysfunction generally, they may also be more sensitive to neurological changes, as there are two separate ways in which neurological dysfunction can alter ERP amplitude.

## 5. Conclusions

In this study we observed cross-sectional differences in the N100, P300 and N400 responses of downhill mountain bike athletes associated with age, sex and caffeine consumption. Females had higher amplitude N100 and P300 responses, while the amplitude of the N400 was lower in older participants. N100 amplitudes and latencies were smaller in those reporting more than normal caffeine consumption. Furthermore, neurological processing performance was consistent between downhill mountain bike athletes and collision sport athletes reported previously. The individual differences observed in this study suggest that demographic factors and caffeine consumption should be considered when applying ERP evaluations to assess SRC- and RHI-related differences in neurological processing in downhill mountain bikers. Future investigations should quantify RHI exposure in downhill mountain bikers to investigate the association between RHI exposure and ERPs in this population. Furthermore, baseline assessment of ERPs may be necessary for post-injury ERPs to be of clinical utility due to the capacity for demographic factors to alter responses. Future research should investigate whether these same factors influence post-injury assessments of ERPs to further inform the clinical utility of N100, P300 and N400 ERPs.

## Figures and Tables

**Table 1:** Demographic and lifestyle factors among downhill mountain bike athletes.

		Males (*n* = 66)	Females (*n* = 26)
Age [Mean (95% CI)]		24.8 (23.07, 26.54)	24.41 (21.61, 27.2)
Caffeinated beverages consumed	*None*	24	9
	*1–2*	35	17
	*3–4*	7	0
*Difference From Normal*	*Less*	12	6
	*Same*	40	18
	*More*	10	2
*Mood*	*Very Low*	2	1
	*Low*	4	2
	*Good*	44	18
	*Very Good*	16	5
*Alcohol Consumption (previous 24 h)*	*No*	20	45
	*Yes*	6	21
*Nicotine Consumption (previous 24 h)*	*No*	24	61
	*Yes*	2	5
*Psychoactive Consumption (previous 24 h)*	*No*	25	60
	*Yes*	1	6

**Table 2:** Mean and 95% Confidence Intervals (CIs) of benchmark Event-Related Potential (ERP) profile in downhill mountain bike athletes.

	Males (*n* = 66)		Females (*n* = 26)	
ERP Outcome	Mean	95% CI	Mean	95% CI
N100 Amplitude (μV)	4.49	(4.02, 4.96)	5.64	(4.67, 6.61)
N100 Latency (ms)	97.66	(93.71, 101.61)	94.54	(87.98, 101.1)
P300 Amplitude (μV)	6.02	(5.31, 6.73)	7.93	(6.53, 9.32)
P300 Latency (ms)	275.36	(266.83, 283.9)	276.46	(262.08, 290.63)
N400 Amplitude (μV)	3.16	(2.83, 3.49)	3.04	(2.44, 3.63)
N400 Latency (ms)	414.15	(399.76, 428.54)	430.08	(400.59, 459.57)

**Table 3:** Multivariable comparison of age and sex (reference: Male) with Benchmark Event-Related Potentials (ERPs).

ERP Outcome	Exposure	Estimate (95% CI)	*p* Value
N100 Amplitude	Sex	**1.15 (0.20, 2.10)**	**0.019**
	Age	−0.01 (−0.07, 0.06)	0.827
N100 Latency	Sex	−3.1 (−10.53, 4.33)	0.409
	Age	0.09 (−0.39, 0.58)	0.704
P300 Amplitude	Sex	**1.89 (0.48, 3.29)**	**0.009**
	Age	−0.05 (−0.14, 0.04)	0.255
P300 Latency	Sex	0.93 (−15.12, 16.99)	0.908
	Age	−0.41 (−1.45, 0.63)	0.434
N400 Amplitude	Sex	−0.15 (−0.75, 0.46)	0.631
	Age	**−0.06 (−0.10, −0.02)**	**0.003**
N400 Latency	Sex	16.04 (−13.05, 45.13)	0.276
	Age	0.29 (−1.59, 2.18)	0.759

**Table 4:** Exploratory Bivariate comparisons of N100, P300 and N400 Event-Related Potentials with caffeine and alcohol consumption (no consumption as the reference).

		Estimate (95% CI)	*p* Value
N100 Amplitude (μV)	Caffeine (Y/N)	−0.10 (−1.02, 0.82)	0.834
	*More*	**−1.39 (−2.68, −0.09)**	**0.036**
	*Less*	0.38 (−0.72, 1.48)	0.491
N100 Latency (ms)	Caffeine (Y/N)	−2.60 (−9.55, 4.36)	0.46
	*More*	**−18.96 (−28.29, −9.64)**	**<0.001**
	*Less*	−4.35 (−12.27, 3.57)	0.278
P300 Amplitude (μV)	Caffeine (Y/N)	−0.26 (−1.63, 1.11)	0.707
	*More*	−0.32 (−2.29, 1.66)	0.751
	*Less*	1.24 (−0.44, 2.92)	0.146
P300 Latency (ms)	Caffeine (Y/N)	−7.41 (−22.36, 7.55)	0.328
	*More*	−5.78 (−27.53, 15.97)	0.599
	*Less*	9.28 (−9.19, 27.74)	0.321
N400 Amplitude (μV)	Caffeine (Y/N)	−0.20 (−0.79, 0.40)	0.514
	*More*	−0.40 (−1.26, 0.46)	0.353
	*Less*	−0.40 (−1.13, 0.33)	0.275
N400 Latency (ms)	Caffeine	1.02 (−26.32, 28.35)	0.941
	*More*	20.91 (−18.72, 60.53)	0.297
	*Less*	−1.70 (−35.34, 31.94)	0.92

## Data Availability

Data will be made available upon reasonable request.

## Supplementary Materials

The following supporting information can be downloaded at: https://www.mdpi.com/article/10.3390/s25175388/s1, Table S1: Bivariate association (estimates and 95% Confidence Intervals [95%CI]) between exposures and Event-Related Potential outcomes.

## Abbreviations

The following abbreviations are used in this manuscript:

SRC	Sport-Related Concussion
EEG	Electroencephalogrpahy
ERP	Event-Related Potentials
RHI	Repetitive Head Impacts
