# Supplementary material for: Neurophysiology of Downhill Mountain Bike Athletes—Benchmark Assessments of Event-Related Potentials
Source: Sensors (Basel). 2025 Sep 1;25(17):5388. doi: 10.3390/s25175388 (PMC12431017; doi:10.3390/s25175388)
Supplement: Supplementary file 1 [file sensors-25-05388-s001.zip › sensors-3788115-supplementary.pdf]

Supplementary Table S1. Bivariate association (estimates and 95% Confidence Intervals [95%CI]) between exposures and Event-Related Potential outcomes.

		N100 Amplitude (µV)		N100 Latency (ms)		P300 Amplitude (µV)	
**Exposure**	**Level**	**Estimate (95%CI)**	**p value**	**Estimate (95%CI)**	**p value**	**Estimate (95%CI)**	**p value**
Mood	Very Low	0.67 (-1.92, 3.26)	0.607	9.20 (-10.58, 28.98)	0.358	2.64 (-1.22, 6.50)	0.178
	Low	1.02 (-0.93, 2.96)	0.301	-0.47 (-15.34, 14.40)	0.95	0.90 (-2.00, 3.79)	0.541
	Good	-0.43 (-1.5, 0.65)	0.432	-3.38 (-11.60, 4.83)	0.416	-0.47 (-2.05, 1.11)	0.558
	Very Good	ref		ref		ref	
Alcohol	Some	-0.04 (-1.02, 0.93)	0.931	2.91 (-4.49, 10.30)	0.437	-0.25 (-1.69, 1.20)	0.735
	None	ref		ref		ref	
Nicotine	Some	-0.11 (-1.77, 1.54)	0.893	0.71 (-11.87, 13.30)	0.91	0.88 (-1.60, 3.35)	0.484
	None	ref		ref		ref	
Psychoactives	Some	-0.29 (-1.95, 1.36)	0.727	2.57 (-10.00, 15.14)	0.685	1.31 (-1.16, 3.78)	0.295
	None	ref		ref		ref	

		P300 Latency (ms)		N400 Amplitude (µV)		N400 Latency (ms)	
**Exposure**		**Estimate (95%CI)**	**p value**	**Estimate (95%CI)**	**p value**	**Estimate (95%CI)**	**p value**
Mood		2.64 (-1.22, 6.50)	0.955	6.50 (0.18, -1.24)	0.513	-1.24 (-44.35, 41.87)	0.333
		0.90 (-2.00, 3.79)	0.606	3.79 (0.54, 8.43)	0.151	8.43 (-23.90, 40.76)	0.941
		-0.47 (-2.05, 1.11)	0.748	1.11 (0.56, 2.86)	0.293	2.86 (-14.78, 20.49)	0.532
		ref		ref		ref	
Alcohol		7.38 (-8.38, 23.14)	0.355	-0.05 (-0.68, 0.58)	0.878	-17.91 (-46.46, 10.64)	0.216
		ref		ref		ref	
Nicotine		-9.23 (-36.36, 17.90)	0.501	0.12 (-0.95, 1.19)	0.822	-11.84 (-61.23, 37.55)	0.635
		ref		ref		ref	
Psychoactives		-1.50 (-28.70, 25.69)	0.913	-0.44 (-1.52, 0.63)	0.413	-46.78 (-95.25, 1.69)	0.058
		ref		ref		ref	
